# Advances in mucopolysaccharidosis research: the impact of mass spectrometry-based approaches

**DOI:** 10.1186/s12014-025-09562-4

**Published:** 2025-11-24

**Authors:** Madan Gopal Ramarajan, Kishore Garapati, Vivek Ghose, Akhilesh Pandey

**Affiliations:** 1 Manipal Academy of Higher Education (MAHE), Manipal, Karnataka India; 2 Institute of Bioinformatics, International Technology Park, Bangalore, Karnataka India; 3 Department of Laboratory Medicine and Pathology, Mayo Clinic, 200 First Street SW, Rochester, MN 55905 USA; 4 Center for Individualized Medicine, Mayo Clinic, Rochester, MN USA

**Keywords:** Mass spectrometry, Mucopolysaccharidoses, LC-MS/MS, Glycosaminoglycans, Proteomics, Metabolomics, Glycomics, Biomarkers, Enzyme replacement therapy, Gene therapy

## Abstract

Glycosaminoglycans (GAGs) are linear polysaccharide chains that are usually linked to proteins to create proteoglycans and play an essential role in the extracellular matrix (ECM). Mucopolysaccharidoses (MPS) are a group of rare disorders that arise due to impairment in the breakdown of glycosaminoglycans (GAGs). Key technological advances in mass spectrometry (MS) have had a significant impact on the study and diagnosis of MPS, as well as its clinical management. This review summarizes the current applications of mass spectrometry-based approaches in MPS, emphasizing its role in the understanding of pathophysiological disease mechanisms, and towards improved patient care. Mass spectrometry-based proteomics and metabolomics have identified novel biomarkers and metabolic perturbations related to the pathophysiology of MPS. In addition, mass spectrometry-based glycomics analyses have been employed for the structural characterization of GAGs to reveal their heterogeneity. The sensitivity and specificity of liquid chromatography tandem mass spectrometry (LC-MS/MS) as compared to conventional methods for the quantitation of GAGs have revolutionized diagnostics. High-resolution mass spectrometers such as Orbitrap and Fourier transform ion cyclotron resonance, permit more accurate GAG characterization. Mass spectrometry has also proven valuable in monitoring patients undergoing treatment, thereby allowing the sensitive monitoring of the therapeutic efficacy of both enzyme replacement and gene therapies. Mass spectrometry has enabled improved newborn screening and multiplex assays for screening multiple MPS types. Despite the important contributions of mass spectrometry to enhance MPS research and clinical management, there still remain challenges related to long and complex sample preparation processes, lack of standardization and lack of accessibility in routine clinical settings. We envision that future initiatives will incorporate multiple omics technologies to obtain a more holistic view of the pathophysiology of MPS. Fortunately, mass spectrometry technologies and methods continue to evolve rapidly, promising further advancements in MPS diagnosis, monitoring of patients on therapy and research that should ultimately lead to improved patient outcomes and quality of life.

## Introduction

Proteoglycans (PGs) are essential components of almost all tissues, and are found both extracellularly and within cells [1]. They consist of a protein core attached to the glycosaminoglycan (GAG) chains [2, 3]. GAGs are linear, negatively charged polysaccharides composed of repeating disaccharide units, usually an amino sugar and a hexuronic acid. They vary by monosaccharide type and sulfation, resulting in categories such as hyaluronan (HA), chondroitin sulfate (CS), dermatan sulfate (DS), heparin, heparan sulfate (HS), and keratan sulfate (KS) [4]. Unlike other GAGs, KS contains galactose and lacks uronic acid, whereas HA lacks a protein core and is synthesized at the intracellular surface of cell membranes [5]. Mucopolysaccharidoses (MPS) are rare, progressive genetic disorders with multi-organ involvement and shortened life expectancy, resulting from lysosomal enzyme deficits that impair glycosaminoglycan (GAG) breakdown [6, 7]. These diseases are classified as lysosomal storage disorders (LSDs), characterized by the accumulation of substrates within lysosomes. MPS is classified into 11 types based on the presence or absence of defective enzymes and the type of stored GAGs (Table 1) [8]. Accumulation of GAGs in cells and tissues results in a progressive decline in organ function that affects multiple systems, including the central nervous system and musculoskeletal system [9]. These clinical phenotypes differ according to the specific type of MPS, but some overlapping presentations are observed, such as organomegaly, dysostosis multiplex, coarse facies, and neurological symptoms [6, 7, 10, 11].

**Table 1:** MPS types, accumulated GAGs, and associated enzyme defects

MPS type	Major GAGs accumulated in cells/tissues	Deficient enzyme
MPS I (Hurler, Hurler-Scheie, Scheie)	DS, HS	Alpha-L‐iduronidase (IDUA)
MPS II (Hunter)	DS, HS	Iduronate sulfatase (IDS)
MPS IIIA (Sanfillipo A)	HS	N-sulphoglucosamine sulphohydrolase (SGSH)
MPS IIIB (Sanfillipo B)	HS	Alpha-N-acetylglucosaminidase (NAGLU)
MPS IIIC (Sanfillipo C)	HS	Heparan acetyl-CoA: alpha-glucosaminide N-acetyltransferase (HGSNAT)
MPS IIID (Sanfillipo D)	HS	N-acetylglucosamine 6‐sulfatase (GNS)
MPS IVA (Morquio A)	KS, CS	N-acetylgalactosamine 6‐ sulfatase (GALNS)
MPS IVB (Morquio B)	KS	β-galactosidase (GLB1)
MPS VI (Maroteaux-Lamy)	DS	Arylsulfatase B (ARSB)
MPS VII (Sly)	DS, HS, CS	β-glucuronidase (GUSB)
MPS IX (Natowicz)	Hyaluronan	Hyaluronidase (HYAL)
MPS X	DS	Arylsulfatase K

DS: Dermatan sulfate, HS: Heparan sulfate, KS: Keratan sulfate, CS: Chondroitin sulfate

MPS has a worldwide incidence of 1.53–1.56 per 100,000 live births [12]. In the United States, the incidence is estimated at 0.98 per 100,000 live births, with a prevalence of 2.67 per 1 million [13]. In the US, between 1995 and 2015, 721 patients with MPS were registered with the National MPS Society, with 681 patients appropriately classified according to MPS type. As of 2015, Ultragenyx had identified and listed 21 patients with MPS VII in their US registry program. The International Registry for MPS IVA recorded 87 patients in the US between 1998 and 2006 [14]. The combined incidence of all MPS types was 0.98 per 100,000 live births. MPS I, II, and III had the highest incidence at 0.26 per 100,000 live births, whereas MPS IV, VI, and VII had incidences of 0.14, 0.04, and 0.027 per 100,000 live births, respectively [14]. MPS II is the most prevalent subtype in East Asian countries, while MPS III and IV are more common throughout Europe. The prevalence of MPS is influenced by several factors, including ethnic background, founder effects, and regional marriage practices [15].

Owing to its diverse population and various societal factors, India is expected to experience a high prevalence of LSDs. In 2015, the Indian Council of Medical Research (ICMR) and the Department of Health Research (DHR) established a task force to explore the prevalence, molecular spectrum, and phenotype-genotype correlation of various LSDs in the Indian population. The task force identified prevalent LSDs, founder variants of certain storage disorders, and the molecular spectrum of various LSDs in India [13]. The most commonly identified disorders were glycolipid storage disorders (48%), MPS type IV (Morquio disease, 22%), and defective sulfatide degradation (14%) [12, 13, 15]. The study identified 32 adult patients with LSDs out of a total of 2,102 LSD cases. The most common LSD in the adult population is Gaucher disease (37.5%), followed by Fabry disease (13%), MPS IVA and MPS I (10%), and Pompe disease (9%) [15].

Newborn screening (NBS) is a promising method for early diagnosis, allowing the detection of MPS early in life, often before symptoms appear. Reliable screening programs enhance our understanding of MPS epidemiology and facilitate its early detection and treatment [16]. Early diagnosis and treatment are essential for improving patient outcomes [2, 3]. Patients with MPS experience a significantly lower health-related quality of life, influenced by disease severity, diagnosis timing, pain, cognitive issues, mobility, and treatment initiation, with scores below the median reference population in all dimensions [17]. Hematopoietic stem cell transplantation and enzyme replacement therapy (ERT) are currently available therapeutic modalities, with novel approaches under investigation [18].

**Fig. 1:**
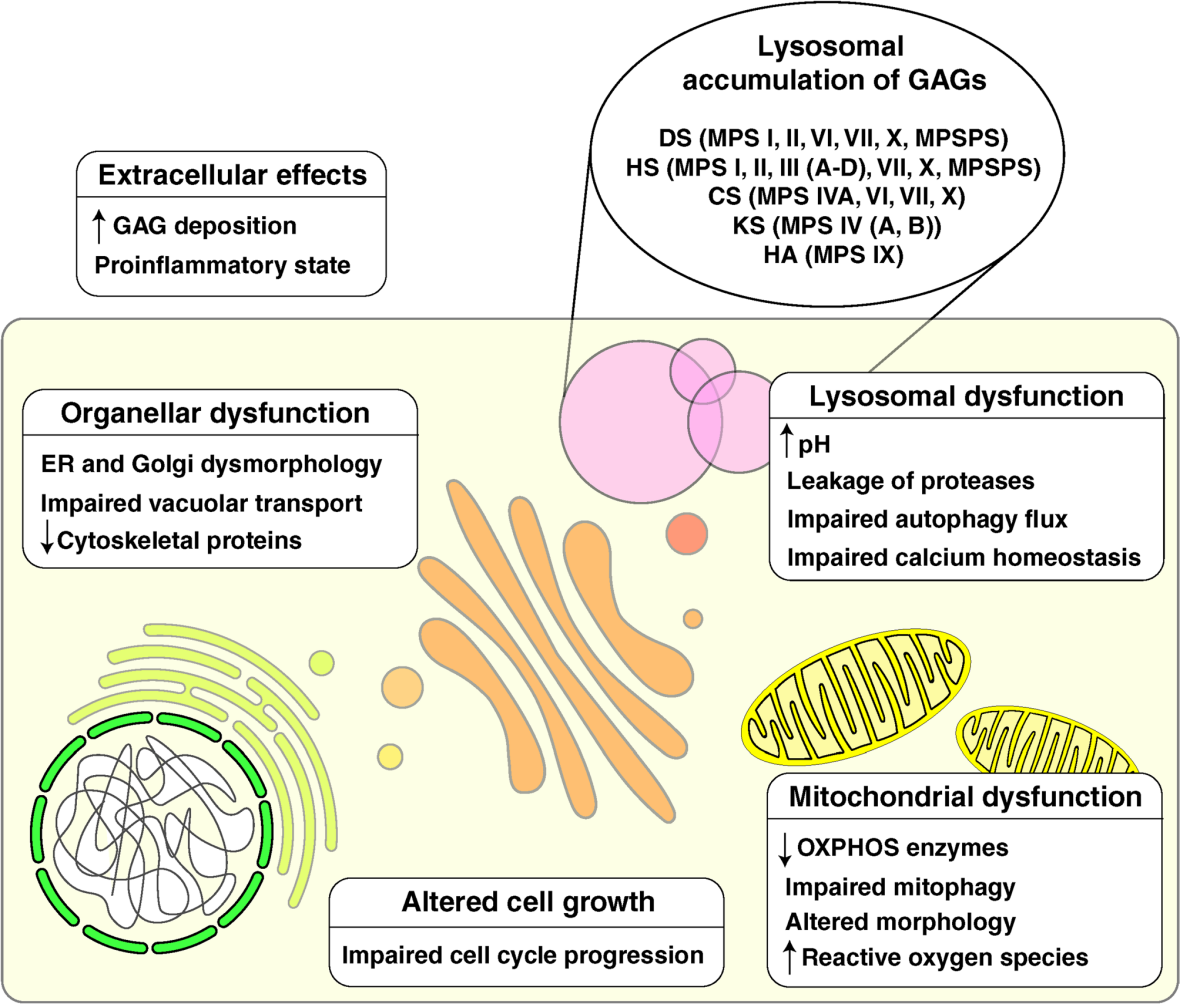
Overview of altered cellular processes in mucopolysaccharidoses (MPS). The accumulation of glycosaminoglycan (GAG) in various MPS disorders is associated with impaired cellular processes. DS: dermatan sulfate; HS: heparan sulfate; CS: chondroitin sulfate; KS: keratan sulfate; HA: hyaluronic acid; MPSPS: mucopolysaccharidosis plus syndrome; ER: endoplasmic reticulum; OXPHOS: oxidative phosphorylation

Lysosomal enzyme deficiency in MPS has functional consequences not only for the lysosome but also for several other cellular compartments and has been reviewed in detail elsewhere [19–21]. Briefly, impaired breakdown and consequent accumulation of GAGs lead to lysosomal stress, which results in altered lysosomal membrane permeability and calcium homeostasis, along with an increase in pH [22]. Autophagy, an essential cellular process in which lysosomes play a central role, has been shown to be impaired or activated in different MPS [23–25]. Apoptotic processes are also affected, as evidenced by an increase in apoptotic markers in certain MPS disease models, although the underlying mechanisms are not clear [26–28]. The downstream effects of lysosomal dysfunction on MPS have been described in several other cellular compartments and processes [20]. Notably, MPS disease models have revealed impaired vacuolar transport function along with ER and Golgi dysmorphology [29, 30]. Studies have also shown mitochondrial oxidative phosphorylation (OXPHOS) enzyme dysfunction along with energy impairment and structural changes in the mitochondria [31, 32]. Research conducted in animal models has indicated that the cytoskeletal machinery is also affected, although the mechanisms are not clear [33, 34]. In addition, further studies have revealed impaired cell growth and proliferation in MPS, with studies showing decreased progression to the S and G2/M phases of the cell cycle [35, 36]. The accumulation of GAGs in the extracellular space results in increased secretion of cytokines, which contribute to a pro-inflammatory state [37, 38]. An illustration of the cellular effects of GAG accumulation on MPS is shown in Fig. 1.

**Fig. 2:**
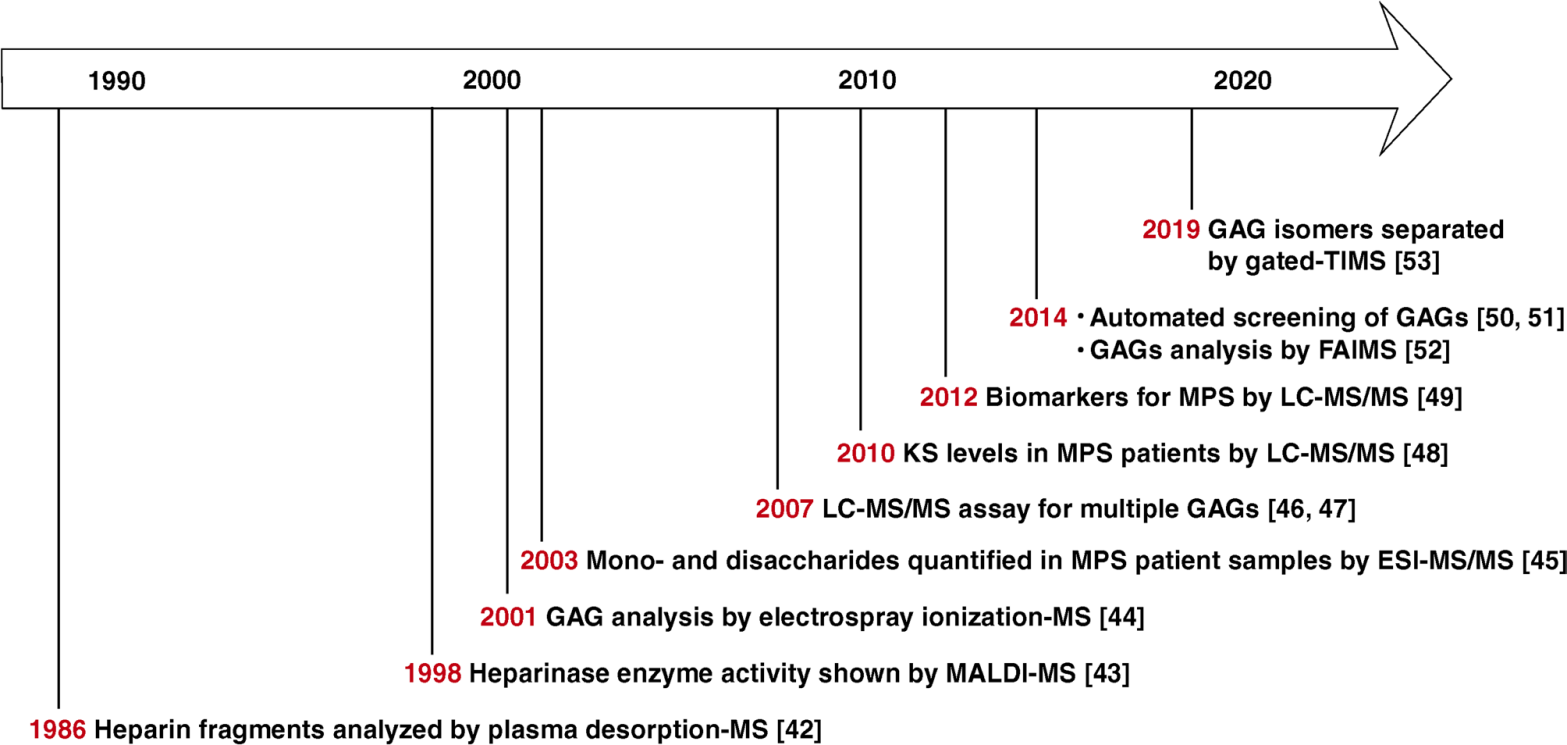
Timeline of mass spectrometry application in mucopolysaccharidoses (MPS) and glycosaminoglycan (GAG) analysis. An overview of important events in the application of various mass spectrometry methods in studying and characterizing GAGs as well as their applications to MPS research. MS: mass spectrometry; MALDI: matrix-assisted laser desorption/ionization; ESI: electrospray ionization; LC-MS/MS: liquid chromatography–tandem mass spectrometry; KS: keratan sulfate; FAIMS: field asymmetric waveform ion mobility spectrometry; TIMS: trapped ion mobility spectrometry

Tandem mass spectrometry (MS/MS) has emerged as a valuable tool in MPS research, enabling screening, early diagnosis, and treatment evaluation through detection of GAGs [39]. MS/MS has revolutionized the diagnosis of LSDs. MS/MS enables highly sensitive and specific analysis of various biomarkers, including urinary GAGs, oligosaccharides, sphingolipids, plasma oxysterols, and lysosphingolipids from dried blood spots [40]. This technology has improved newborn screening for LSDs by allowing high-throughput enzyme activity assays in dried blood samples. Recent advancements in multiplexed MS/MS techniques have further enhanced the simultaneous detection of multiple LSDs in a single assay [41]. MS analysis has been applied to better understand GAG biology and MPS disorders for decades (Fig. 2). In one of the earliest mass spectrometry (MS) applications for GAG analysis published in 1986, McNeal et al. applied plasma desorption–mass spectrometry to analyze heparin-derived disaccharides to reveal structural clues [42]. In 1998, Rhomberg et al. deployed matrix-assisted laser desorption ion mass spectrometry (MALDI-MS) to demonstrate the mechanism of action of heparinase II as a non-random enzyme that catalyzes the depolymerization of heparin-like glycosaminoglycans [43]. Furthermore, in 2001, Zaia and Costello used electrospray ionization-MS (ESI-MS) to analyze complex mixtures of GAGs and deduce their monosaccharide composition using MS/MS by collision-induced dissociation (CID) [44]. Subsequently, in 2003, Ramsay et al. described a method for quantifying mono- and disaccharides in MPS patient samples using ESI-MS/MS [45]. Assays for the targeted analysis of multiple GAGs were developed by Oguma and colleagues using multiple reaction monitoring and deployed for the analysis of MPS patient samples [46, 47]. Further, in 2010, Tomatsu and colleagues developed and validated an LC-MS/MS-based assay for the quantitation of keratan sulfate levels in patients with MPS type IVA (Morquio A disease) [48]. Auray-Blais et al. subsequently developed a urine filter paper-based assay for the quantification of dermatan sulfate and heparan sulfate [49]. In 2014, researchers published reports on the development of automated mass spectrometry-based methods for the analysis of GAGs and their application for the screening and monitoring of MPS [50, 51]. In the same year, Kailemia et al. successfully deployed ion mobility technology (high-field asymmetric waveform ion mobility spectrometry, FAIMS) to separate isomeric and isobaric GAGs and analyzed them using MS/MS [52]. More recently, in 2019, Wei et al. deployed gated-trapped ion mobility spectrometry (TIMS) to resolve highly sulfated GAGs [53]. The timeline depicted in Fig. 2 presents a historical review of the evolution of MS-based assays for the detection of GAGs.

This review aims to summarize the current status and provide a future perspective of mass spectrometry-based approaches in mucopolysaccharidoses research, diagnosis, and management. We have emphasized the role of MS-based omics approaches in understanding MPS pathophysiology and biomarker discovery. In addition to reviewing how MS can aid in better diagnostics and newborn screening, this review also discusses applications of MS for monitoring response to treatment. Finally, we discuss the challenges ahead in combining MS with other omics technologies to promote personalized medicine in patients with MPS.

## Applications of mass spectrometry in MPS clinical research

### Proteomics

**Fig. 3:**
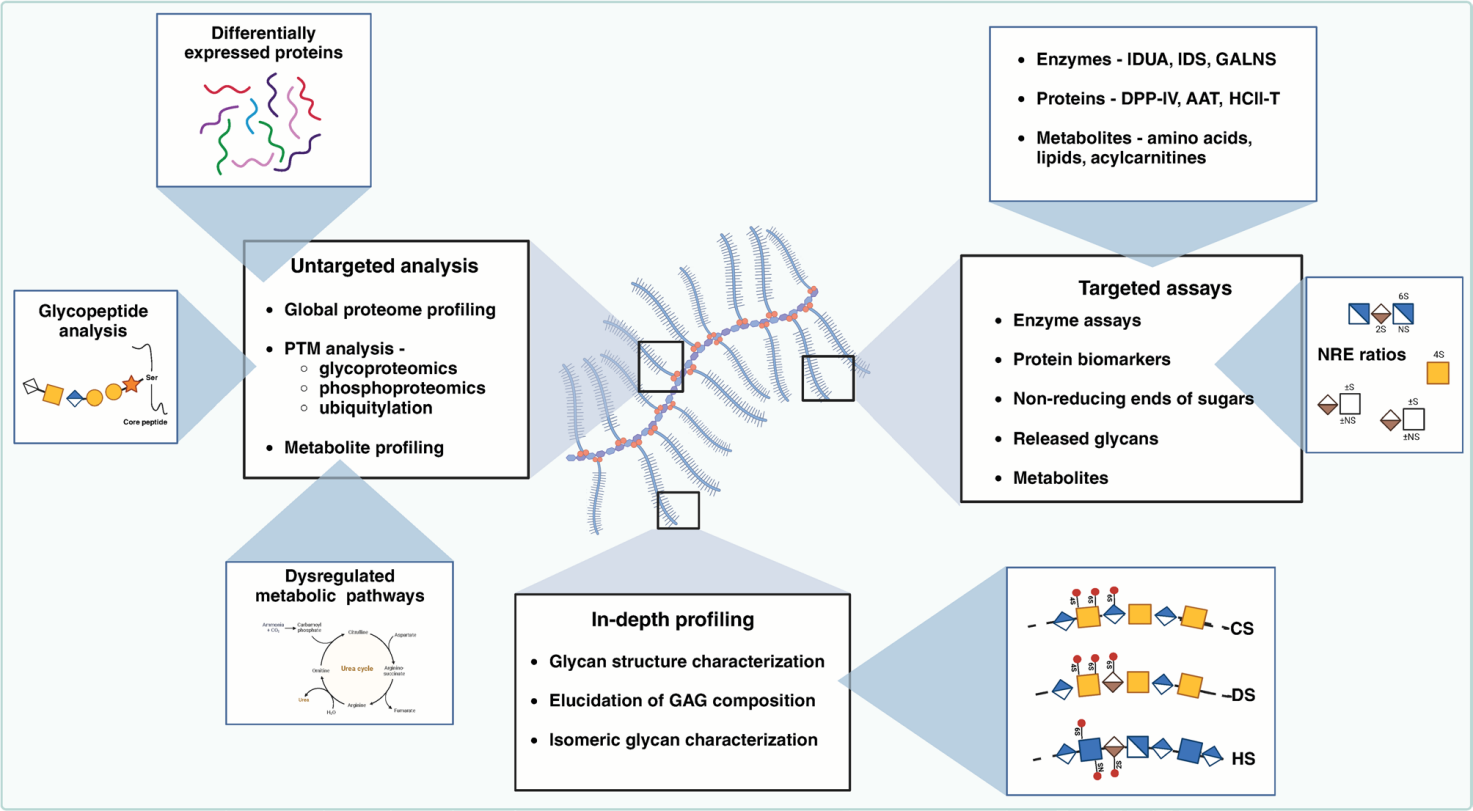
Integrative approaches using mass spectrometry (MS) for comprehensive proteomic, glycomic and metabolomic profiling of mucopolysaccharidoses (MPS). Use of MS for untargeted and targeted analysis of mucopolysaccharidoses (MPS), focusing on GAGs and metabolic pathways. This integrative picture shows how mass spectrometry systems, from untargeted global analysis to very specific targeted assays, can help investigate the complicated biology of MPS. Proteomic and glycomic analyses, biomarker development, and therapy monitoring improve our understanding of disease causes and treatment responses. IDUA: alpha-L-iduronidase; IDS: iduronate-2-sulfatase; GALNS: N-acetylgalactosamine 6‐ sulfatase; DPP-IV: dipeptidyl peptidase – IV; AAT: alpha-1-antitrypsin; HCII-T: heparin cofactor II-thrombin complex; CS: chondroitin sulfate; DS: dermatan sulfate; HS: heparan sulfate

Proteomics, or large-scale study of proteins in the human body, has led to numerous discoveries leading to a better understanding of the basic disease mechanisms and molecular underpinnings of MPS [54–57]. MS-based proteomics methods have been instrumental in detecting, measuring, and analyzing the enzymes involved in, as well as examining alterations in the expression of other proteins associated with MPS (Fig. 3) [58, 59]. The combination of liquid chromatography and tandem mass spectrometry (LC-MS/MS) offers high-resolution analysis of proteins found in biological specimens such as blood and tissue, thereby providing valuable insights for both basic and translational clinical research (Table 2) [60, 61]. Despite the development of numerous proteomics technologies, one-dimensional and two-dimensional polyacrylamide gel electrophoresis (1D SDS-PAGE, 2D SDS-PAGE) remain among the most widely utilized methods in diagnosis [62, 63]. These methods have been used in the past to study LSDs, including MPS, for biomarker detection [58, 64, 65]. However, 2D SDS-PAGE suffers from several limitations owing to the limited resolution of large proteins, high dynamic range of protein abundance, and biological variation inherent to human samples [66, 67]. MS-based proteomics allows accurate identification and quantitation of proteins as well as their post-translational modifications (PTMs) in a high-throughput manner [68–72]. MS offers several advantages, including relative quantitation, and has emerged as an approach with great potential for detecting protein biomarkers of diseases [59]. Recently, by applying stable isotope labeling and bimodal distribution analysis, researchers have successfully identified a set of lysosomal proteins with high confidence in cell lines [73]. A comparison of lysosomal proteomes across multiple cell lines revealed potential novel lysosomal proteins, and researchers confirmed the lysosomal localization of six of these candidate proteins [73]. The six lysosomal proteins are NDFIP2, SLC31A1, SLC12A9, TM7SF3, TMEM63B and TSPAN3. Of these, NDFIP2 facilitates WW binding activity, and numerous E3 ubiquitin ligases possess WW domains, which are responsible for recruiting and ubiquitinating specific target proteins [74]. Proteins such as SLC31A1, SLC12A9, TM7SF3, TMEM63B and TSPAN3 are involved in lysosomal transport and membrane integrity [73]. In a recent study, Pierzynowska et al. [75] reported alterations in proteasome composition and activity in cultured fibroblasts derived from MPS patients, demonstrating genistein’s role in modulating proteasomal activity. There are limited number of studies that have investigated the proteasome in the context of mucopolysaccharidosis (MPS) disorders. The ubiquitin-proteasome system remains largely unexplored within the realm of lysosomal storage disorders, such as MPS.

**Table 2:** Proteomic and metabolomic biomarkers in MPS and their clinical implications

	Biomarker	Sample type	Assay methods	Clinical implications	References
1.	ACTA1, ACTN4, TUBB4B, DNM1, STXBP1, ATP6V1B2, RYR3	Brain tissue from MPS I and control mice	(2D-PAGE) and nano LC-MS/MS	Proteins involved in • Metabolism • Neurotransmission • Cytoskeleton • Biomarkers useful for diagnosis or therapeutic studies	[34]
2.	Protein to creatinine (Cr) ratio: AAT/Cr, GM2A/Cr, L-PGDS/Cr	Urine	MALDI-TOF/TOF	• Better understanding of the pathogenic mechanism of MPSII • Discovery of early diagnostic markers	[58]
3.	FABP, COMP, NID1, COL12A1, GLB1, HEG1, IGFBP7	Urine	LC-MS/MS and MRM-LC-MS/MS	• Urine markers of MPS I, II, and VI • Indicate disease severity • Markers differentiate between neurological and non-neurological MPS II • Aid in early diagnosis and treatment efficacy assessment	[59]
4.	HCII-T	Serum	Immunoassay	• Screening of MPS I and II • Therapeutic monitoring, • Assess disease severity	[76, 77, 170]
5.	DPP4	Serum	SELDI-TOF-MS	• Therapeutic monitoring	[78, 79]
6.	Products of GNS	DBS	LC-MS/MS	• Screening and diagnosis of MPS-IIID	[171]
7.	GAA and IDUA	DBS and buccal swabs	Immuno-SRM	• DBS and buccal swab - a rapid and viable matrix for screening the newborns	[172]
8.	Five lysosomal enzymes: IDUA for MPS I, IDS for MPS II, NAGLU for MPS IIIB, GALNS for MPS IVA, and ARSB(MPS VI) GAG disaccharides: DS, HS0S, HSNS, monoKS and diKS	DBS	LC-MS/MS (MRM mode)	A 2-tier approach • 5-plex enzyme assay using ESI-MS/MS • GAG disaccharides by LC-MS/MS analysis in NBS for MPS and • Reduces false positives	[173]
9.	Enzymatic products of 10 relevant enzymes associated with MPS: IDUA, IDS, SGSH, NAGLU, HGSNAT, GNS, GALNS, GLB1, ARSB, GUSB	DBS	LC-MS/MS	• Single assay for the detection of all the MPS (except MPS IX)	[162]
10.	IDUA and HS, DS	DBS	LC-MS/MS	• Second tier testing of GAGs for MPS confirmed positive with enzyme assay	[174]
11.	IDUA, IDS, NAGLU, GALNS, and ARSB	DBS	LC-MS/MS	• Validation of a 5-plex assay to detect and distinguish MPS I, MPS II, MPS IIIB, MPS IVA, and MPS VI	[175]
12.	LTF, CORO1A, GANAB, and VTN	Leukocytes	LC-MS/MS and SWATH-MS	• Potential biomarkers in MPS IVA for evaluation of bone disease severity ERT	[176]
13.	GalNAc4S or GalNAc6S, N,N-dimethylarginine, Glutamylhydroxyproline, Creatine, Dihydrophaseic acid, N2-Succinyl-L-glutamic acid 5- semialdehyde, N-gamma-Glutamyl-S-allylcysteine, Glutamic acid, Phenylalanylhydroxyproline, Glycylproline, Isoleucylhydroxyproline	Urine	LC-MS/MS	12 potential candidate biomarkers for use in • Screening • Diagnostic • Evaluating the efficacy of enzyme replacement therapy (ERT)	[102]
14.	Carnitine, oleic acid, prolyl-lysine, tetrahydrocorticosterone, arginine, phenlalanylalanine followed by targeted GAG analytes	Urine	LC-MS/MS	Metabolic phenotyping in MPS I • Novel therapeutic targets to counteract the underlying oxidative stress	[103]
15.	Metabolites related to arginine-proline, histidine, and glutathione pathways	Urine	UHPLC with ion mobility and high-resolution mass spectrometer	Functional snapshot profile of metabolites in MPS VI using • Untargeted metabolomics and • Interactions with dermatan sulfate metabolism	[98]
16.	N-acetylserotonin, N-succinyl-L, L-2,6-diaminopimelate, 3-2-Hydroxylphenylpropanoic acid and octanoylglucuronide	Urine	For untargeted metabolite analysis: Ultraperformance liquid chromatography–ion mobility mass spectrometer For targeted metabolite analysis: LC-MS/MS	Metabolic remodeling in subtypes of MPS III using • Untargeted metabolomics • Integrated pathway analysis	[101]
17.	Untargeted metabolites: amino acids, carbohydrates, neurotransmitters, peptides, lipids, nucleotides	Serum	LC-MS/MS	• Metabolic disturbances in MPS III disorders seen during the early stages of the disease progression	[100]

Dermatan sulfate (DS), heparan sulfate no sulfation (HS0S), heparan sulfate N (HSNS), monosulfated keratan sulfate (monoKS), disulfated keratan sulfate (diKS), keratan sulfate (KS), selected reaction monitoring (SRM)

It is known that serum heparin cofactor II-thrombin complex (HCII-T) levels are markedly elevated in untreated MPS I, II, and VI patients, correlating with disease severity and treatment response, making it a valuable biomarker for assessment and monitoring [76, 77]. HCII-T is most effective for dermatan sulfate-storing MPS but may also be informative for heparan sulfate-storing diseases [77]. Despite decreasing with treatment, HCII-T levels may not fully normalize, particularly in severe cases [77]. Serum dipeptidyl peptidase-IV (DPP-IV) activity can be used to screen for early MPS. In patients with MPS, serum DPP-IV activity, urinary GAG to creatinine (GAG/Cre) ratio, total ADA, and ADA-1 isoenzyme activity were significantly higher than those in healthy controls [78]. The urinary GAG/Cre ratio and serum DPP-IV activity showed excellent diagnostic accuracy in differentiating patients with MPS from healthy controls [78]. Dipeptidyl peptidase IV (DPP-IV) is a potential biomarker of MPS that can be used to monitor treatment efficacy. Beesley et al., utilized surface enhanced laser desorption/ionization time of flight (SELDI-TOF) mass spectrometry to study differences in plasma protein levels, particularly apolipoprotein CI (ApoCI), between MPS patients and healthy controls [79]. The ratio of truncated ApoCI (ApoCI’) to full-length ApoCI was altered in patients with MPS due to increased DPP-IV activity. The activity of DPP-IV was reduced in patients with MPS I, who were receiving enzyme replacement therapy, suggesting that it could serve as a biomarker for evaluating treatment efficacy [79, 80].

In patients with MPS, alterations in serum matrix metalloproteinases (MMP) may function as biomarkers for diagnosis, monitoring, and treatment response [81]. Patients with MPS III exhibited significantly lower MMP-9 activity and protein levels and higher MMP-2 levels than controls. Patients with MPS II showed significantly elevated proMMP-2 activity and MMP-2 protein levels. In patients with MPS VI, enzyme replacement therapy decreased MMP-9 activity and protein levels for up to four months post-treatment [81]. Targeted proteomics performed on urine samples obtained from patients with MPS I, II, and VI showed increased expression of cartilage oligomeric matrix protein, insulin-like growth factor-binding protein 7, and beta-galactosidase in MPS II with neurological phenotype compared with MPS II with non-neurological phenotype [59]. The observed potential to distinguish patients by severity of their condition suggests that these markers may determine disease severity at the time of the initial assessment of the patient [59]. Liu et al. performed simultaneous analysis of serum and urine proteomes to identify differentially abundant proteins in MPS I [82]. Five proteins were identified that were altered in the serum and urine of patients with MPS I, with CD163, SHBG, and APOA1 significantly changed in both matrices [82]. Proteomic technologies are continuing to be developed and will likely increase our understanding of MPS, in addition to other lysosomal storage diseases, as we continue in the omics era. Further, MS-based analyses of different PTMs contribute to our understanding of disease processes in MPS, as outlined below.

#### Phosphoproteomics

Phosphoproteomics allows researchers to identify and quantify the phosphorylation events that regulate various cellular processes, including those related to lysosomal function. For instance, the presence of hyperphosphorylated tau in the medial entorhinal cortex and dentate gyrus of the MPS III B mouse model has provided key evidence of neurodegeneration [83, 84]. Phopshoproteomics of fibroblasts derived from Hurler-Scheie syndrome patients showed significant alterations in proteins involved in cytoskeletal changes, cellular dysfunction and apoptosis [85]. Comparative proteomic and phosphoproteomic analyses of the hippocampus in a Niemann-Pick type C1 mouse model provided insights into disease mechanisms. Mutant animals exhibit increased phosphorylation of T286 on CaMKIIα and S1303 on NR2B, which is essential for learning, memory, and potentially inducing neuronal death, even in late disease stages [86]. Elucidating the pattern of phosphorylation of GAG-degrading enzymes can provide insights into novel prognosis/diagnostic biomarkers, as well as therapeutic targets.

#### Glycoproteomics

It has become clear from recent progress in the glycoproteomic field that GAGs and proteoglycans are highly complex, filled with unexpected subtleties and potential clinical opportunities (Fig. 3) [87–92]. Recently, MS-based glycoproteomics has proven to be crucial for the elucidation of GAG structure and function in complex patterns, similar to those found in chondroitin sulfate (CS) linkage regions [90, 91, 93]. Novel methods have revealed new levels of complexity in CS structures and identified novel proteoglycans, as well as unexpected sites and compositions of some forms [93–95]. This research is of clinical importance as it could lead to the discovery of novel biomarkers, such as the glycosaminoglycan linkage region from urinary bikunin for β3GalT6-deficient spondylodysplastic Ehlers-Danlos syndrome [96]. Nikpour et al. have shown the need for comprehensive site-specific glycosylation analysis to identify disease-related markers and design molecular-targeted therapies [52]. Glycoproteomics approaches to understand the intricately regulated pathways of GAG synthesis can pinpoint targets for intervention with substrate reduction therapy (SRT). In general, glycoproteomics is an excellent technology for understanding the off-target effects of SRT, which may help improve therapeutic strategies. Examining the interaction of GAGs with proteins will lead to the identification of novel sites for SRT, which selectively interfere with deleterious MPS-mediated interactions [97]. An increasing body of research in this area indicates that to fully utilize these molecules in basic and clinical research, a comprehensive understanding of proteoglycan characterization, GAG-mediated interactions, and GAG biosynthesis regulation is critical.

### Metabolomics

Metabolomics has become a useful tool for investigating and treating MPS with an abundance of information concerning metabolic changes characteristic of this group of rare genetic diseases [20]. Metabolomics has helped elucidate the biochemical basis of MPS through detailed analysis of the metabolic profiles of patients with MPS (Table 2) [98]. For example, targeted metabolomics of heart and liver tissues from mouse models of MPS IIIB revealed an increase in the levels of branched-chain amino acids (BCAA), free carnitines and acylcarnitines indicating dysregulation in BCAA and fatty acid catabolism [99]. Fu et al. studied serum metabolomics in MPS III A and MPS IIIB patients and found elevated levels of metabolites from lipid derivatives and cysteine metabolites consistent with oxidative stress and inflammation. Further, there was a significant decrease in the metabolite levels of key amino acids, neurotransmitter pathways, and neuroprotective compounds [100]. Tebani et al., studied a French cohort of 49 patients diagnosed with MPS III and its subtypes and reported metabolic changes observed in MPS III through untargeted and targeted metabolomics [101]. The urea cycle and arginine-proline metabolism were the two pathways that were altered. Upregulation of these metabolic pathways has been associated with high autophagic activity and glucose reduction, consistent with their involvement in the bioenergetic balance [101]. In addition, metabolic phenotyping of patients with MPS VI has revealed dysregulation of arginine-proline, histidine, and glutathione metabolism [98]. Recent research utilizing untargeted metabolic profiling of urine samples from different types of MPS patients (including untreated and those treated with ERT) revealed increased levels of 12 metabolites across all types of MPS [102]. These 12 metabolites belong to acylaminosugars, dipeptides, amino acids and their derivatives [102]. This metabolic dysfunction arises from downstream consequences triggered by increased accumulation of GAGs in lysosomes, as evidenced by an untargeted analysis [103]. In addition, recent studies have demonstrated accumulation of metabolites such as gangliosides including globotriaosylceramide (Gb3) and lactosylceramide (LacCer), especially in neuronopathic forms of MPS [104–106]. Elevated levels of LysoGb3 have been observed in MPS I, II and III patients [107]. Recent studies on mice, strongly argue that gene therapy can correct metabolic derangements at the cellular level and re-establish balance, leading to improved clinical outcomes in mucopolysaccharidoses when integrated with metabolomics [108, 109]. Currently available human metabolomics data also represent a promising approach with the potential to identify and refine candidate therapeutics that may help treat this complex group of disorders [110].

### Glycomics

Glycomic analyses provide a biological guide to the molecular composition and functions of GAG structures and are not limited to sulfation patterns and chain lengths. Techniques such as MS are commonly employed to elucidate the structural features of GAGs [111]. This is particularly important for GAGs that have complex and heterogeneous structures. Standardized kits for ultra-high-performance liquid chromatography (UHPLC) with MS/MS enable simultaneous analysis of multiple GAG disaccharides, facilitating increased sensitivity and potential clinical use. This approach combines UHPLC with mass spectrometry to achieve rapid high-throughput analysis [112]. The mass spectrometry-based glycomics test is useful for distinguishing between different MPS subtypes [113]. In addition to the disaccharides, free oligosaccharides in the urine have also been employed in evaluation of MPS, such as uronic acid subunits (MPS I, II, IIIA and VII), n-acetylhexosamine (MPS IIIB), and n-acetylneuraminic acid (MPS IVA) [114, 115]. Such assessments accurately differentiate between various MPS subtypes, thereby serving as a highly predictive diagnostic method for quick biochemical diagnosis by analyzing urine GAG profiles [113, 116]. Mass spectrometry is a powerful tool for assessing the efficacy of therapy and natural progress over time in patients with MPS with dynamic GAG profiles [39, 117, 118]. Matrix-assisted laser desorption ionization time-of-flight (MALDI-TOF) mass spectrometry has emerged as a sensitive technique for structural analysis of GAGs and their fragments [119, 120]. MALDI-TOF based assays have been able to identify diagnostic free oligosaccharide patterns for certain MPS and their subtypes (MPS I, II, IIIA, IVA, IVB) [120–123]. Although the current mass-spectrometry-based glycomics approaches provide many benefits, some obstacles still need to be overcome: standardization of methods and implementation of artificial intelligence techniques in data analysis and interpretation [124–126]. Developing complete glycan libraries and cutting-edge computational tools will help identify limitations of current workflows and lead to enhanced accuracy of glycan analysis in the future.

## Biochemical analysis of GAGs

### Integration with chromatographic methods

Several major breakthroughs in the analysis of GAGs have been made possible by liquid chromatography-tandem mass spectrometry (LC-MS/MS) technologies, which allow the evaluation of GAG levels in different biological matrices, especially for mucopolysaccharidoses [46, 47, 127]. Implementing these methods has enabled the sensitive and specific measurement of disaccharides derived from various GAGs, such as chondroitin sulfate (CS), dermatan sulfate (DS), heparan sulfate (HS), and keratan sulfate (KS). MS-based methods offer advantages over traditional techniques including dye-specific assays, chromatography and electrophoresis in terms of sensitivity, specificity, and throughput [51, 117].

### Detection of GAGs by tandem mass spectrometry

**Fig. 4:**
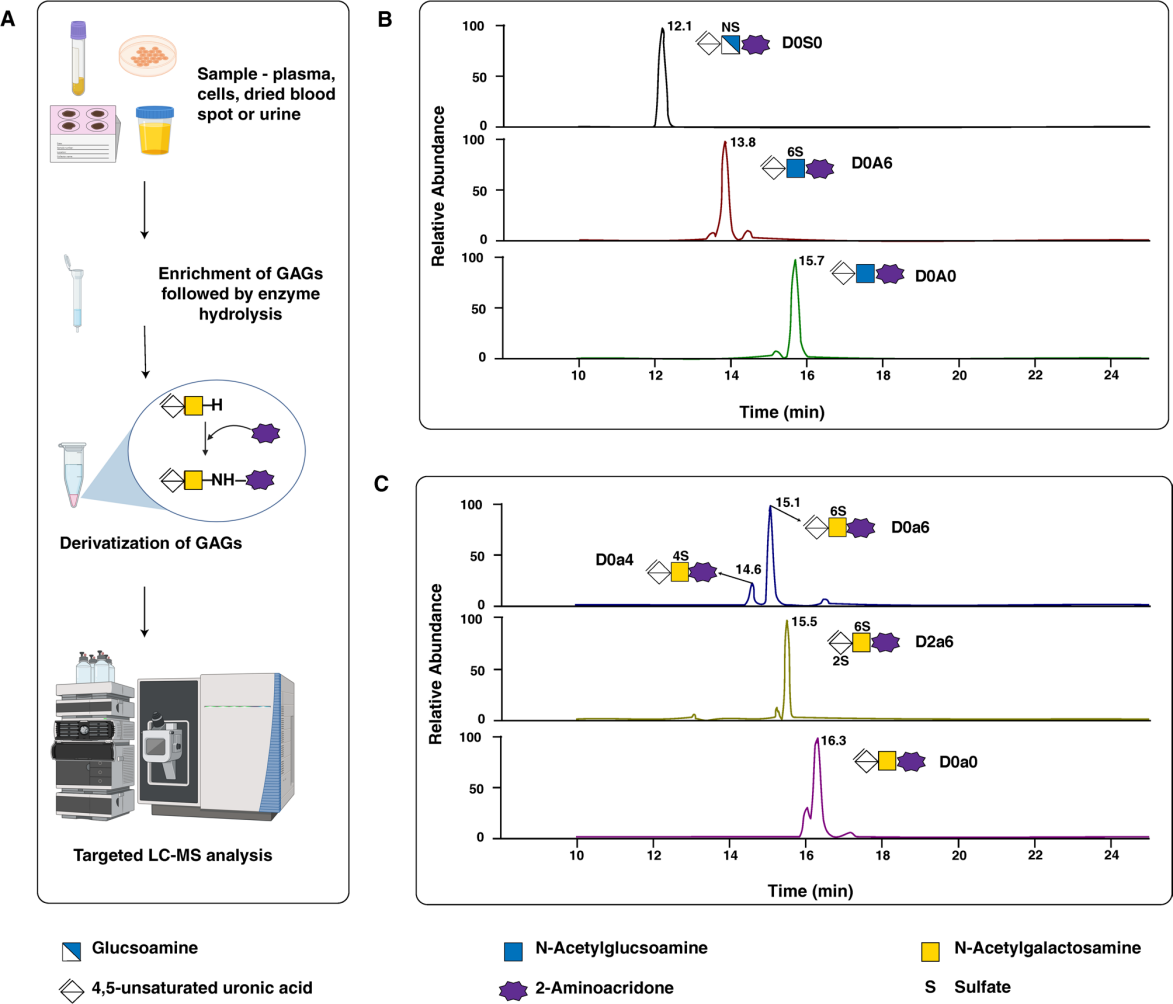
Released disaccharide analysis using mass spectrometry-based assay. (**A**) Workflow depicts enrichment of glycosaminoglycans (using strong anion exchange columns from different sample matrices, followed by enzyme hydrolysis to reduce linear polymers to disaccharide units. These disaccharide units are derivatized by reductive amination followed by analysis on the mass spectrometer. (**B**) Total ion chromatograms of different forms of heparan sulfate disaccharide comprising of hexuronic acid and hexosamine or N-acetyl-D-hexosamine linked by β(1,4) bond (D0S0: ΔUA-GlcNS; D0A6: ΔUA-GlcNAc6S and D0A0: ΔUA-GlcNAc). (**C**) Total ion chromatograms of different forms of chondroitin sulfate disaccharide comprising of hexuronic acid and N-acetyl-D-hexosamine linked by β(1,3) bond (D0a0: ΔUA-GalNAc; D0a4: ΔUA-GlcNAc4S; D0a6: ΔUA-GlcNAc6S; D2a6: ΔUA2S-GlcNAc6S). The disaccharides depicted in B and C were identified from standards subjected to enzyme hydrolysis followed by derivatization with 2-aminoacridone. (ΔUA- 4,5-unsaturated uronic acid; GlcN: Glucosamine; GlcNAc: N-acetylglucosamine; GalNAc: N-acetylgalactosamine; S: sulfate)

Numerous MS/MS protocols for GAG quantification have been reported, covering sulfated GAGs in cell lines, urinary GAGs, mono- and disaccharides in tissue extracts, plasma/serum or urinary GAGs, normal human cartilage GAGs, and GAGs from dried blood spots (Table 3) [47, 111, 118, 128–132]. Most widely accepted glycomics approach for evaluation of GAGs include protocols based on enzymatic digestion followed by disaccharide analysis [129, 133, 134]. The analysis of GAGs after enzymatic cleavage into mono- or di-saccharides is based on several key factors such as the structural complexity and heterogeneity of GAGs. By breaking them down into smaller units, it allows for precise structural characterization, which is difficult when analyzing intact macromolecules. Furthermore, the analysis of disaccharides enables improved resolution and quantification using LC-MS/MS. In 2001, Oguma et al. developed an ESI mass spectrometry protocol for quantifying HS and KS in serum and plasma, which was later refined to include DS and adapted for dried blood spots (DBS) [46, 127]. Polysaccharides were enzymatically digested with heparinase, keratanase, and chondroitinase B to release HS, KS, and DS, respectively, with disaccharides detected by LC-MS/MS [46, 47]. In 2014, Osago et al. introduced a comprehensive LC-MS/MS method for the simultaneous analysis of disaccharides from all four GAG classes and identified and quantified 23 disaccharides, including di- and tri-sulfated species [135]. Enzymatic digestion with chondroitinase ABC, hyaluronidase, heparinase, and keratanase was used to characterize the GAG composition in articular cartilage [135]. Disaccharides with identical molecular masses but different structures were separated on a porous graphitized carbon column and identified using selected reaction monitoring (SRM) transitions with the same Q1 but different Q3 specific to each disaccharide, thus distinguishing isomers within and between classes [135]. Lawrence et al., proposed a method to detect non-reducing ends of GAGs, where enzyme deficiencies in MPS diseases result in polysaccharide accumulation with unique non-reducing ends [136]. Bacterial enzyme digestion and reductive amination with fluorescent tags followed by LC-MS/MS are used to quantify these sugars (Fig. 4) [134, 136]. An acid-catalyzed methanolysis process using methanolic hydrochloric acid was developed for the UPLC-MS/MS analysis of individual GAGs [49, 137]. Methanolysis produces desulfated and derivatized disaccharides, among other oligosaccharides, with MS/MS used for DS-, CS-, HS-, and KS-related disaccharide quantification after chromatographic separation [128]. This method has been applied to various sources, including urine, cerebrospinal fluid (CSF), and animal tissues, for high-risk screening, diagnosis, and therapy assessment [39, 128, 131, 138–142].

**Table 3:** List of LC-MS/MS-based assays available to quantify glycosaminoglycans (GAGs) in screening for MPS disorders (since 2010)

	Analytical method	Sample Type	GAGs/Biomarkers analyzed	Clinical utility	Advantages	References
1.	LC-MS/MS	Plasma, Serum	DS, HS, KS	Early screening, assessment of clinical course, and therapy efficacy	High sensitivity and specificity; useful for multiple MPS types	[46]
2.	LC-MS/MS (MRM mode)	DBS	DS, HS0S, HSNS, monoKS and diKS	A 2-tier approach of 5-plex enzyme assay followed by LC-MS/MS analysis of GAGs broadens the range of NBS for MPS	Reduces false positives	[173]
3.	LC-MS/MS	DBS	HS-0 S, HS-NS, and DS	Measurement of GAGs in combination with enzyme assays allows effective discrimination of affected patients from the non-affected population	Reduces false positives	[177]
4.	nanoLC-MS/MS	Urine	GAG-NRE profiling of HS, DS and CS	GAG-NRE profiling using 2-aminobenzamide reductive amination of GAGs, increases the sensitivity of the LC-MS/MS analysis for MPS types IH/IS, II, IIIc, IVa and VI	Faster turnaround time, sensitive, semi-quantitative using internal disaccharides	[111]
5.	MS/MS (Direct injection)	Plasma, Serum, DBS	HS, DS, CS, and KS	High throughput mass spectrometry assay. Diagnosis of MPS II, IVA, and IVB and therapeutic monitoring of various MPS	This technique is more valuable for rapid screening; however, it cannot separate ΔDi-4 S, ΔDi-6 S, and ΔDiHS-6 S, that have same molecular weight.	[117]
6.	LC-MS/MS	Plasma, Serum, urine	HS, CS, DS, monosulfated KS, disulfated KS and its ratio to total keratan sulfate	Diagnosis of MPS I, MPS II, III, IVA, IVB, and VI, and therapeutic monitoring of various MPS	Measurement of GAG levels in blood and urine useful for diagnosis of MPS	[118]
7.	ESI-MS	Cells, tissues and fluids from affected individuals, dogs and mice	NREs from HS, DS and CS	Qualitative and Quantitative assessment of the biomarkers in biological samples.	NREs can serve as biomarkers, which are indicators that can help identify specific MPS disorders	[136]
8.	MS/MS	Fibroblasts, plasma, urine	Disaccharides – HS, DS, CS, HA	Useful for screening MPS types.	Sensitive, fluorescent measurement, stable derivatives, improved detection and resolution	[134]
9.	LC-MS/MS	Urine	CS, DS, HS	Screening and monitoring of MPS patients, particularly for MPS III, MPS IVA, MPS VI, and mild GAG elevations	Reliable, straightforward, specific for GAG species, can monitor therapeutic intervention	[139]
10.	LC-MS/MS	CSF	HS	Useful for assessing experimental therapies	Useful for determining treatment efficacy.	[178]
11.	LC-MS/MS	Urine	DS, HS, KS	Accurate diagnosis of MPS, monitoring ERT efficacy	More sensitive and reliable tool than the DMB ratio for MPS	[179]
12.	LC-ESI-MS/MS.	Fibroblasts cell line, urine, plasma, CSF from MPS I, Mice with MPS I – liver	Trisaccharide biomarker tagged with a fluorescent label in MPS I	Sensitive and effective means of diagnosing MPS I. Potential biomarker for prognosis following therapeutic intervention	Rapid, sensitive and low-cost assay without depolymerization and labelling; Useful for early disease detection, including NBS, disease progression, response to therapy	[180]
13.	LC-MS/MS	Urine	CS, DS, HS, KS	Screening and diagnosis of MPS, monitoring therapy efficacy	Reduces false positives/negatives, accounts for age/physiological conditions, reveals actual status of DS, HS, KS	[181]
14.	LC-MS/MS	Urine	GAG Fragments – sulfated disaccharide, trisaccharide, tetrasaccharide, disulfated pentasaccharide of HS, DS and CS	Improve the diagnostic process by measuring specific glycosaminoglycan fragments for precise diagnosis	Single panel detects and differentiates between all 10 subtypes of MPS Enables precise quantification of unique GAG fragments for longitudinal biochemical monitoring post-therapy.	[182]
15.	Internal disaccharide - hydrophilic-LC-MS/MS	Mice Brain, Cerebrospinal Fluid (CSF)	HS, HS_met_ HS_dig_	Reduction of HS in brain and CSF after treatment with CM-rhSulfamidase.	Useful for assessing treatment efficacy in patients affected with MPS IIIA	[183]
16.	LC-MS/MS	Urine	DS, HS and KS	Sensitive assay, accurate and reliable in quantifying the urinary GAGs	Used as first tier, and those with elevated GAGs will be further evaluated for enzyme activity and genetic testing	[184]
17.	Internal disaccharide - hydrophilic-LC-MS/MS	DBS	D0A0, D0S0, D0a4	Use of second-tier GAG analysis of newborn DBS	Reduce false positives	[185]
18.	Endogenous disaccharide hydrophobic-LC-MS/MS	DBS	UA-HNAc(1 S)	Use of second-tier GAG analysis of newborn DBS	Reduce false positives	[185]
19.	Sensi-pro assay: Glycan Reductive Isotope Labeling (GRIL)-LC/MS for disaccharide profiling of GAGs. Reductive amination with isotopically tagged aniline.	DBS	I0S0, I0S6	Use of second-tier GAG analysis of newborn DBS	Reduce false positives	[185]
20.	Sensi-pro Lite hydrophobic-LC-MS/MS	DBS	I0S0, I0S6	Use of second-tier GAG analysis of newborn DBS	Reduce false positives	[185]
21.	LC-MS/MS	CSF	Disaccharides - HS, DS	The method described is a robust and highly sensitive one that could be used to quantify GAGs in various matrices	Measure individual GAGs species	[161]
22.	LC-MS/MS	DBS	GAG-NRE	Detection of endogenous GAG NRE for MPS-IIIA-D, -IVA, -VI, VII, and GM1 gangliosidosis	Second-tier testing aimed at reducing false positive rate	[186]
23.	LC-MS	Urine	HS/DS ratio and KS/HS ratio	Used to discriminate between MPS type I, II, III, IV, VI, VII	Specific GAG ratios complement the quantified GAGs by LC-MS enhance discrimination of MPS types	[187]
24.	UPLC-MS/MS	Urine	Urinary oligosaccharides and glycoamino acids	One multiplexed assay for diagnosis of subtypes MPS and glycoproteinosis	Diagnosis and subtyping of MPS and Glycoproteinosis	[188]

Heparan sulfate (HS), chondroitin sulfate (CS), dermatan sulfate (DS), heparan sulfate no sulfation (HS0S), heparan sulfate N (HSNS), monosulfated keratan sulfate (monoKS), disulfated keratan sulfate (diKS), keratan sulfate (KS), non-reducing end GAG structures (GAG-NREs), hyaluronan (HA), electrospray ionization (ESI), heparan sulfate metabolites (HS_met_), digests of heparan sulfate after heparinase treatment (HS_dig_)

Disaccharide codes: D0A0: ΔUA-GlcNAc; D0S0: ΔUA-GlcNS; D0a4: ΔUA-GlcNAc4S; I0S0: IdoA-GlcNS; I0S6: IdoA-GlcNS6S

ΔUA- 4,5-unsaturated uronic acid; GlcN: Glucosamine; GlcNAc: N-acetylglucosamine; GalNAc: N-acetylgalactosamine; IdoA: L-Iduronic acid; S: sulfate (Ref: Lawrence et al., 2008 [189])

These approaches provide methods to directly map the composition of large GAG polymers, which are essential for establishing structure-activity relationships and for quality control [89]. Additionally, these improvements, along with advancements in sample preparation, online separation techniques, and automation software for analysis, have greatly improved the throughput and precision of detection and quantification of GAG [143]. Such progress is essential for the detection of GAGs and setting new standards for quality control, thereby accelerating the entry of new compositions into clinical trials [89, 90, 143–145].

### High resolution LC-MS/MS

High resolution LC-MS/MS platforms, such as Orbitrap, Fourier Transform Ion-Cyclotron Resonance (FT-ICR), and high-resolution Time-of-Flight (TOF) mass spectrometers are key technologies for evaluation of GAGs because they permit detailed structural characterization and increased separation of complex mixtures [53, 111, 146, 147]. High resolution – LC-MS/MS delivers superior identification of GAGs due to its high mass accuracy and resolution. The use of ion-mobility separation with electron transfer dissociation has been used to differentiate isobaric species structure elucidation, which is important because of the complexity and heterogeneity of GAGs [53]. High resolution – LC-MS/MS, particularly with Orbitrap technology, offers high mass accuracy and resolution that have proven to be instrumental for the accurate measurement of molecular weight signatures and hence structural analysis of GAGs [147]. This technology can provide accurate mass measurements up to a resolution of 1,000,000 and permits discrimination of many isobaric species within complex mixtures [147, 148]. The combination of HCD and photodissociation techniques coupled with FT-ICR or Orbitrap mass spectrometers allows for detailed analysis of the structural features of complex biomolecules such as GAGs [147, 149, 150]. This integrative approach leverages the best aspects of different fragmentation methods and modeling strategies to yield deeper insights into molecular structures, contributing significantly to biochemical and therapeutic research [151].

MS approaches, such as time-of-flight secondary ion mass spectrometry (ToF-SIMS) and ion mobility coupled with orbitrap mass spectrometry improves isomer resolution and resolves structural features that are not observable by MS alone [146]. ToF-SIMS, can be deployed to decipher the molecular complexity of GAGs [146]. For instance, Hook et al. used ToF-SIMS, along with multivariate analysis, to analyze GAGs at nanogram levels (< 200 ng) and to distinguish 400 different GAG types, within a short time frame (3–4 h) and pharmaceutical-grade heparin produced for therapeutic applications [146]. The methodology demonstrated the capability to differentiate pharmaceutical-grade heparin derived from various animal species and manufacturers with a detection sensitivity of 0.001 wt% [146]. Further, the development of these technologies integrated with next-generation data acquisition systems is evolving to increase the resolution and precision of GAG analysis, allowing more in-depth structural elucidation and identification of unknowns [151].

## Clinical applications of mass spectrometry in MPS - Screening, diagnosis and monitoring

Mass spectrometry has been used for therapeutic monitoring of mucopolysaccharidoses by detecting sulfated mono- and disaccharides in urine and blood, serving as sensitive biomarkers to assess treatment efficacy [52]. More specific biomarkers, such as HS and DS, in plasma and urine remain elevated despite long-term ERT, indicating their higher sensitivity compared to total uGAGs [139]. Additionally, 3D facial imaging and dysmorphometrics have shown promise as noninvasive tools for monitoring treatment response in patients with MPS I [152].

ERT can reduce GAG levels in various tissues but has limitations in addressing brain and skeletal manifestations and mass spectrometry-based methods have been used to quantify HS and DS in tissues and biological fluids [153, 154]. In MPS II mice, HS levels were higher than DS levels, and an 8-week ERT significantly reduced HS and DS levels in all tissues except the brain, with reductions sustained over 16 weeks [154]. Non-reducing end GAG detection methods are more sensitive and specific than traditional dye-based assays for monitoring ERT response in MPS I (Table 4) [155].

**Table 4:** MS-based assays used for therapeutic monitoring following enzyme replacement therapy

MPS type	Sample type	Therapy type	MS technique	Biomarkers monitored	Outcome	References
MPS IIIA	CSF	Treated with CM-rhSulfamidase	LC-MS/MS	HS	HS can be reliably monitored in CSF for the ongoing Phase I clinical trials of the drug	[183]
MPS I	Serum, CSF, urine	ERT with laronidase (Aldurazyme)	LC-MS/MS	CS, DS, HS	Serum concentrations of DS and HS were within reference limits or marginally elevated in patients receiving IV ERT, this method can be used to monitor the GAG levels after initiating ERT	[155]
MPS I	CSF	HSCT in combination with intrathecal (IT) and intravenous (IV) ERT.	LC-MS/MS	CS, DS and HS	The assay has potential in monitoring patients with mucopolysaccharidoses receiving treatments targeted to the brain	[190]
MPS I, II, III	DBS	ERT	LC-MS/MS	LysoGb3—globotriaosylsphingosine	A secondary biomarker that could be used as therapeutic marker for neuronopathic forms of MPS	[107]
MPS VI	Leukocytes	ERT	UPLC-MS/MS	CS and DS	Used as a biomarker for intracellular GAG accumulation	[191]
MPS VII	Urine	Treated With Vestronidase Alfa	LC-MS/MS	DS, CS, HS	Substantial decrease of uGAGs is observed and indicating an improvement in the disease physiology	[192]

CSF: Cerebrospinal fluid; HSCT: Hematopoietic stem cell transplantation; ERT: Enzyme replacement therapy; CS: Chondroitin sulfate; DS: Dermatan sulfate; HS: Heparan sulfate; NRE: Non-reducing end of disaccharides

## Emerging tools – Innovations in instrumentation and automation

Recent advancements in MS technology have significantly improved its application in drug discovery and clinical settings [156]. Automation has been critical for enhancing throughput and for reducing human intervention in LC-MS/MS analyses, making them more suitable for routine clinical use [143]. High-throughput MS techniques have revolutionized drug discovery by enabling the label-free screening of thousands of compounds, offering cost-effective and physiologically relevant assays [157, 158]. Advancements in sample processing, instrumentation, and data acquisition have enhanced the sensitivity and specificity of MS-based proteomics, demonstrating its potential for biomarker discovery and verification [159]. In the field of ADMET profiling, innovations such as automated MS/MS optimization, high-speed LC separation, and integrated software solutions have significantly enhanced the speed and quality of bioanalysis [160]. Emerging technologies for sample introduction, ionization, and mass analysis are expected to further increase throughput and potentially transform existing bioanalytical paradigms [144].

MS methods show high sensitivity and robustness in quantifying HS and DS GAGs in various matrices such as tissues, CSF, and brain cell populations [161]. MS-based assays enabled the detection of significant GAG accumulation in both an MPS II mouse model and CSF from patients with neuronopathic MPS II, including those on enzyme replacement therapy. The exceptional sensitivity of MS-based assays allows for GAG quantification in isolated mouse brain cell populations, highlighting that GAG accumulation is notably higher in microglia than in astrocytes and neurons [161]. A high-throughput LC-MS/MS assay has been developed with ultrahigh sensitivity, enabling GAG measurements from small samples of cerebrospinal fluid and isolated neurons [161]. The increased sensitivity and specificity of this method make it valuable for evaluating the therapeutic effects in MPS disorders. Further, MS-based diagnostics are expanding the clinical landscape, offering rapid and accurate disease detection and therapeutic monitoring [162]. A multiplex LC-MS/MS assay was developed to screen for 10 MPS disorders simultaneously in dried blood spots and fibroblasts, indicating the potential for newborn screening and diagnosis of multiple MPS types [162]. This assay detected 10 enzymatic activities for all MPS disorders, except MPS-IX. Unlike previous methods, this can be used to measure all MPS enzymes, offering a simple, rapid, and precise solution for newborn screening and diagnostic laboratories.

## Challenges and limitations

There are several limitations, including the complexity of GAG structure elucidation in mixtures, challenges in sample preparation, constraints in sensitivity, limitations of analytical methods and a lack of standardization [163]. The identification and measurement of GAGs can be challenging because of the identical masses of several of their components combined with significant structural heterogeneity, which is not limited to sulfation patterns, chain length, and stereochemistry [116, 136, 164]. Isolating and purifying GAGs from various biological matrices without altering their structure and composition can also be challenging [92]. The lack of appropriate standards that encompass all GAG species poses a significant challenge for achieving absolute quantification with high accuracy. Certain GAG types or modified structures are present in very low abundance and are not easily quantifiable by MS, especially in mild cases of MPS, where overall GAG levels may not be significantly elevated, leading to false negatives [165]. Because of the complex nature of GAGs, sufficient structural information may not be obtained by analytical methods, such as collision-induced dissociation (CID), which induces glycosidic cleavages [166–168]. The lack of standardized protocols for GAG analysis using MS leads to variability across laboratories owing to inconsistencies, complicating inter-lab comparison of data and establishment of reliable reference ranges for GAG levels in patients with MPS [117, 129]. Other limitations include cost and access because of relatively expensive MS instrumentation and specific training requirements, which make these techniques less amenable for routine clinical use [53, 126, 164]. Presently available screening assays focus on quantification of released disaccharides from GAGs but do not consider the particular structural variations of specific types of GAGs. Bridging the gap between research findings and translating them into clinical practice requires long-term studies and involves large cohorts. Nevertheless, development of newer and better methodologies along with standardized efforts can mitigate these problems so that the robustness, reproducibility and applicability of MS in clinical MPS management can be improved.

## Future directions

The ongoing integration of advanced omics technologies, especially mass spectrometry-based approaches, is providing a new impetus for advancing precision medicine in patients with MPS. When combined, multi-omic analyses (genomics, transcriptomics and metabolomics) provide a fuller view of pathophysiological alterations, which can be the basis for accurate diagnoses and personalized therapies [101]. Recent high-throughput methods using mass spectrometry have further improved glycosaminoglycan quantification, which has been suggested to improve newborn screening and diagnosis [82, 169]. Together, these advances illustrate the clinical utility of integrating mass spectrometry with other omics technologies to enhance patient care and deliver precision medicine for mucopolysaccharidoses.

## Data Availability

No datasets were generated or analysed during the current study.

## Abbreviations

BCAA: Branched-chain amino acids
CID: Collision-induced dissociation
CS: Chondroitin sulfate
DPP-IV: Dipeptidyl peptidase-IV
DS: Dermatan sulfate
ERT: Enzyme replacement therapy
FAIMS: Field asymmetric waveform ion mobility spectrometry
GAGs: Glycosaminoglycans
HCD: High-energy collision-induced dissociation
HCII-T: Heparin cofactor II-thrombin complex
HS: Heparan sulfate
HSCT: Hematopoietic stem cell transplant
KS: Keratan sulfate
LC-MS/MS: Liquid chromatography-tandem mass spectrometry
LSDs: Lysosomal storage disorders
MMP: Matrix metalloproteinase
MPS: Mucopolysaccharidoses
MS: Mass spectrometry
MS/MS: Tandem mass spectrometry
SDS-PAGE: Sodium dodecyl sulfate polyacrylamide gel electrophoresis
SELDI-TOF: Surface enhanced laser desorption/ionization time of flight mass spectrometry
SRT: Substrate reduction therapy
ToF-SIMS: Time-of-flight secondary ion mass spectrometry
uGAGs: Urinary glycosaminoglycans
UHPLC-MS/MS: Ultra-high-performance liquid chromatography-tandem mass spectrometry
